# Type 2 Autoimmune Hepatitis and Nonadherence to Medication Correlate With Premature Birth and Risk of Postpartum Flare

**DOI:** 10.1002/hep4.1714

**Published:** 2021-05-21

**Authors:** Kathryn Olsen, James Hodson, Vincenzo Ronca, Amber G. Bozward, Jennifer Hayden, Grace Wootton, Matthew Armstrong, David H. Adams, Omar El‐Sherif, James Ferguson, Ellen Knox, Tracey Johnston, Fiona Thompson, Ye Htun Oo

**Affiliations:** ^1^ University Hospitals Birmingham National Health Service Foundation Trust Birmingham United Kingdom; ^2^ Center for Liver and Gastro Research Institute of Immunology and Immunotherapy University of Birmingham Birmingham United Kingdom; ^3^ National Institute for Health Research Birmingham Biomedical Research Center European Reference Network Rare‐Liver Center University Hospitals Birmingham National Health Service Foundation Trust and University of Birmingham Birmingham United Kingdom; ^4^ Birmingham Women’s Hospital Birmingham United Kingdom

## Abstract

Autoimmune hepatitis (AIH) is an immune‐mediated chronic liver disease that affects all ages, including women of childbearing age. Optimal management during pregnancy is poorly defined. We aimed to explore the clinical and biochemical course of AIH in the antenatal and postpartum periods, and assess factors associated with premature birth and postpartum flares. Pregnant women with AIH reviewed in the autoimmune liver disease clinic at the Queen Elizabeth Hospital Birmingham between 2009 and 2020 were identified retrospectively, and clinical, biochemical, and immunological data 1 year before conception to 1 year postpartum were collected. Analysis was performed to identify trends in blood markers over the antenatal period, with an interrupted time series approach used to assess postpartum trends. Data were available for n = 27 pregnancies (n = 20 women), with median gestation of 38 weeks (30% premature) and most having type 1 AIH (78%) and delivering via caesarean section (63%). Levels of alanine transaminase, aspartate transaminase, and immunoglobulin G all declined significantly during gestation, followed by significant step‐change increases after delivery. Postpartum flare developed in 58% of pregnancies. AIH type 2 was associated with a higher rate of premature births (67% vs. 19%, *P* = 0.044), and a trend toward a higher rate of postpartum flare (100% vs. 48%, *P* = 0.053). Although not significant, medication nonadherence was associated with almost double the risk of prematurity (40% vs. 24%, *P* = 0.415) and postpartum flare (80% vs. 44%, *P* = 0.109). *Conclusion:* Biochemical and immunological remission of AIH occurs during pregnancy, although subsequent postpartum flare is common. Type 2 AIH is associated with a higher risk of premature birth and postpartum flare, although further research is required to validate and explain this finding.

**Abbreviations**
AIH: autoimmune hepatitis
ALP: alkaline phosphatase
ALT: alanine transaminase
AST: aspartate transaminase
CI: confidence interval
IgG: immunoglobulin G
INR: international normalized ratio
IQR: interquartile range
LKM: liver‐kidney‐microsome
SLA/LP: soluble liver antigen/liver‐pancreas
ULN: upper limit of normal

Autoimmune hepatitis (AIH) is a chronic, immune‐mediated, progressive liver disease with variable presentation, ranging from asymptomatic to fulminant liver failure.^(^ ^1^ ^)^ Inflammation of the hepatic parenchyma frequently evolves toward fibrosis, cirrhosis, and eventually to liver failure, exposing patients to the complications of cirrhosis and the risk of hepatocellular carcinoma.^(^ ^2^ ^)^ AIH has a prevalence of 16‐18 cases per 100,000 in Europe, with an increasing incidence.^(^ ^3^ ^)^ Although it is well accepted that breakdown in immune tolerance leads to AIH, the pathogenesis of AIH is still largely unknown; it appears to occur in genetically predisposed individuals, triggered by an environmental factor.^(^ ^4^ ^)^

AIH can affect any age group, including women of childbearing age.^(^ ^2^, ^5^ ^)^ Oligomenorrhea is common in patients with active disease or established cirrhosis. Nonetheless, many women with well‐controlled disease can conceive and undergo pregnancies without complications.^(^ ^6^ ^)^ With improvements in the quality of care of AIH, pregnancies are becoming more frequent.^(^ ^5^, ^7^ ^)^ The optimal management of AIH during pregnancy is an immunosuppressive regimen with azathioprine +/‐ prednisolone, but avoiding mycophenolate, due to its teratogenic effects.^(^ ^2^ ^)^

During pregnancy, the course of the disease generally follows a pattern of remission.^(^ ^4^, ^7^, ^8^, ^9^ ^)^ Mild episodes of AIH flares can occur during the first trimester, but more severe flares are described postpartum.^(^ ^7^, ^8^, ^10^, ^11^ ^)^ AIH can be associated with an increased risk of preterm births, having a low‐birth‐weight baby, and occurrences of liver decompensation during pregnancy.^(^ ^8^ ^)^ Additionally, obstetric risks increase in those with liver cirrhosis, with the most commonly observed complication being variceal bleeding.^(^ ^6^ ^)^ Further risk factors for pregnancy in AIH have been suggested, including the presence of soluble liver antigen/liver‐pancreas (SLA/LP) and Sjögren’s syndrome–related antigen A (Ro/SSA) antibodies, poor disease control in the year preceding pregnancy, and an absence of immunosuppression during pregnancy.^(^ ^8^, ^11^ ^)^

The aims of this study were to assess maternal and fetal outcomes in patients with AIH, with a particular interest in postpartum flare and premature birth. In addition, the study aimed to quantify biochemical and immunological changes occurring during the antenatal and postpartum periods.

## Materials and Methods

This is a retrospective study that included all pregnant women with AIH seen in the dedicated autoimmune outpatient clinic in the Liver Transplant Unit and Center for Rare Disease (European Reference Network Rare Liver Center) at Queen Elizabeth Hospital Birmingham (England) between January 2009 and September 2019, with follow‐up until July 2020. The diagnosis of AIH was made on the basis of the International Autoimmune Hepatitis Group’s simplified criteria.^(^ ^12^ ^)^ Liver cirrhosis was diagnosed by liver biopsy or ultrasound findings. AIH subtype was classified based on the presence of associated antibodies; type I AIH is associated with antinuclear antibodies (ANAs) and/or anti‐smooth antibodies, and type II AIH with anti‐liver‐kidney‐microsome‐1 (LKM‐1) antibodies.^(^ ^1^ ^)^ Patients with overlap syndrome (i.e., AIH with primary sclerosing cholangitis or primary biliary cholangitis), and pregnancies occurring in liver‐transplant recipients, were excluded. Only pregnancies that resulted in a live birth were included in the analysis.

Data relating to patient demographics, disease‐related factors, preconception medication regimes, and pregnancy outcomes were extracted from electronic patient records, with ethics approval. Premature birth was defined as a birth occurring before 37 weeks’ gestation. Any evidence of medication nonadherence reported in the electronic patient records were also noted, defined as patients stopping medications or reducing doses against or without medical advice in the year before conception, or during pregnancy. Results of blood tests (alanine transaminase [ALT], alkaline phosphatase [ALP], aspartate transaminase [AST], bilirubin, immunoglobulin G [IgG], albumin, and international normalized ratio [INR]) taken as part of routine care during the antenatal period (from conception to birth) and the postpartum period (from birth to 12 months after delivery) were recorded. Based on these tests, antenatal/postpartum flares were defined based on one of the following criteria, as described previously^(^ ^7^, ^8^ ^)^:

- ALT *or* AST greater than 2 times the upper limit of normal (ULN); or
- ALT *or* AST greater than 1.5 times ULN *and* IgG greater than 1.5 times ULN *and* presence of typical symptoms of a disease flare (e.g., jaundice, abdominal pain, fatigue).

In the case of antenatal flares, these criteria had to be met between conception and birth; patients who already met these criteria before conception required a further rise in blood markers to be classified as an antenatal flare. For postpartum flares, the criteria had to be met within 12 months after delivery, with patients having antenatal flares at the time of delivery requiring a further rise in blood markers to be classified as a postpartum flare.

### Statistical Analysis

Initially, associations between pregnancy outcomes and a range of variables were assessed. Nominal variables were reported as percentages and analyzed using Fisher’s exact test. Continuous variables were reported as mean ± SD, or as medians and interquartile ranges (IQRs), with comparisons performed using Mann‐Whitney U tests.

Trends in blood markers over the antenatal period were then assessed. Before this analysis, the distributions of each marker were assessed graphically, with log_10_ transformations applied where positive skew was detected. General linear models were then produced, with a blood marker set as the dependent variable, and the gestation time when the sample was collected set as a covariate. The pregnancy ID was additionally included as a covariate in the models, allowing each pregnancy to have a separate intercept, to account for baseline variability. The goodness of fit of the models was then assessed by examination of the residuals. Where poor fit was detected, additional terms were added to the model to improve the fit. In models where the dependent variable had been log_10_‐transformed, the resulting coefficients were then antilogged, and converted into percentage changes per month.

Models were then produced to assess postpartum trends, and to compare these to the antenatal data, using an interrupted time series approach. General linear models were produced that contained three covariates, the first of which were the pregnancy ID and the timing of the sample, relative to delivery. A binary factor, stating whether the sample was antenatal or postpartum, was also included, to identify any step changes occurring directly after delivery. In addition, an interaction term between the timing of the sample and the variable stating whether it was collected in the antenatal or postpartum period was also added to the model. This allowed for separate gradients in the antenatal and postpartum periods, and produced a *P* value comparing them. All analyses were performed using IBM SPSS 22 (IBM Corp., Armonk, NY), with *P* < 0.05 deemed to be indicative of statistical significance throughout.

## Results

### Demographics

After excluding one still birth and two miscarriages, 27 pregnancies in 20 women were included in the study, with 5 women contributing two pregnancies and 1 woman contributing three pregnancies to the cohort. The analysis was performed at a pregnancy level, and demographics of the cohort are reported in Tables 1 and 2. The mean age at conception was 29 years, and most of the cohort was of White ethnicity (63%). Twenty‐one pregnancies (78%) occurred in women with type 1 AIH, with the remaining 6 (22%) in women with type 2 AIH; conception was a median of 6 years after AIH diagnosis. Twelve pregnancies (44%) occurred in women with cirrhosis, 10 (37%) in women with fibrosis, and 5 (19%) in women with no fibrosis or cirrhosis. Most of the pregnancies occurred in the absence of additional autoimmune conditions. Where patients did have additional autoimmune conditions, these were type 1 diabetes mellitus, systemic lupus erythematosus, celiac disease, alopecia, and immune thrombocytopenia. ANAs were present in 56% of pregnancies, anti‐smooth muscle antibodies in 52%, and Ro‐52 antibodies in 50% (not mutually exclusive).

**TABLE 1:** Demographics of the Cohort

	Total (n)	Statistic
Age at conception (years)	27	29 ± 5
Ethnicity (% White)	27	17 (63%)
Twin pregnancy	27	1 (4%)
Previous pregnancies	27	
*None*		14 (52%)
*1‐2*		9 (33%)
*>2*		4 (15%)
Previous pregnancy complications*	13	
*None*		9 (69%)
*Miscarriage*		2 (15%)
*Stillbirth*		2 (15%)
Diabetes before pregnancy	27	
*No*		23 (85%)
*Type 1*		2 (7%)
*Type 2 (steroid induced)*		2 (7%)
AIH diagnosis to conception (years)	27	6 (3‐10)
AIH type	27	
*Type 1*		21 (78%)
*Type 2*		6 (22%)
Associated autoimmune disease^†^	27	7 (26%)
Fibrosis/cirrhosis	27	
*None*		5 (19%)
*Mild fibrosis*		2 (7%)
*Moderate fibrosis*		7 (26%)
*Advanced fibrosis*		1 (4%)
*Cirrhosis*		12 (44%)
Prepregnancy immunosuppression regimes	27	
*No immunosuppression*		2 (7%)
*Single immunosuppression*		10 (37%)
*Dual immunosuppression*		14 (52%)
*Triple immunosuppression*		1 (4%)
Prepregnancy immunosuppression agents		
Azathioprine	27	18 (67%)
Prednisolone	27	16 (59%)
UDCA	27	3 (11%)
Mercaptopurine	27	3 (11%)
Budesonide	27	2 (7%)
Mycophenolate^‡^	27	1 (4%)
Tacrolimus	27	1 (4%)
Rituximab^§^	27	1 (4%)
Medication nonadherence	27	10 (37%)
ANA (% positive)	27	15 (56%)
ANA pattern	25	
*Negative*		12 (48%)
*Homogenous*		7 (28%)
*Speckled*		5 (20%)
*Mixed*		1 (4%)
Anti‐smooth muscle antibodies (% positive)	27	14 (52%)
LC‐1 antibodies (% positive)	24	1 (4%)
SLA/LP antibodies (% positive)	24	7 (29%)
Ro‐52 antibodies (% positive)	12	6 (50%)
f‐actin antibodies (% Positive)	12	2 (17%)
LKM blot (% positive)	24	6 (25%)
LKM antibodies (% positive)	26	5 (19%)

Note: Data are reported as n (%), median (IQR), or mean ± SD, as applicable.

* For the subgroup of patients with previous pregnancies.

^†^ Other than type 1 diabetes mellitus.

^‡^ Stopped at 10 weeks’ gestation.

^§^ Given as a one‐off dose in the 6 months before conception.

Abbreviations: f‐actin, filamentous actin; and LC‐1, liver cytosol antigen type‐1; UDCA, ursodeoxycholic acid.

**TABLE 2:** Details of Pregnancies

Pregnancy No.	Premature Birth*	AIH Subtype	Cirrhosis/Fibrosis	Medication Nonadherence Concerns	Antenatal Flare	Postpartum Flare	Delivery Method
1	No	1	Cirrhosis	No	No	Yes	Vaginal
2	No	1	Cirrhosis	No	No	No	Vaginal
3	No	1	Cirrhosis	No	No	No	Caesarean
4	No	1	None	No	No	No	Caesarean
5	Yes	1	None	No	N/A	Yes	Caesarean
6	No	1	None	No	No	Yes	N/A
7	No	1	Mild fibrosis	No	No	No	Caesarean
8	No	1	Moderate fibrosis	No	No	No	Vaginal
9	No	2	Cirrhosis	Yes	No	Yes	Caesarean
10	Yes	2	Cirrhosis	Yes	No	Yes	Caesarean
11	No	1	Moderate fibrosis	No	No	Yes	Vaginal
12	No	1	Moderate fibrosis	No	No	Yes	Vaginal
13	Yes	1	Moderate fibrosis	No	No	No	Caesarean
14	No	1	Moderate fibrosis	No	No	No	Vaginal
15	No	1	Cirrhosis	No	Yes	Yes	Caesarean
16	Yes	2	Moderate fibrosis	No	No	Yes	N/A
17	Yes	2	Cirrhosis	Yes	Yes	Yes	N/A
18	No	1	Cirrhosis	Yes	No	Yes	Caesarean
19	Yes	1	Cirrhosis	Yes	Yes	Yes	Caesarean
20	No	2	Cirrhosis	Yes	No	Yes	Vaginal
21	No	1	Moderate fibrosis	No	No	No	Caesarean
22	No	1	Advanced fibrosis	Yes	No	Yes	Vaginal
23	No	1	Mild fibrosis	Yes	No	No	Vaginal
24	No	1	Cirrhosis	Yes	No	No	Caesarean
25	Yes	2	Cirrhosis	No	N/A	N/A	Caesarean
26	No	1	None	No	No	No	Caesarean
27	Yes	1	None	Yes	No	Yes	Caesarean

* Birth occurring before 37 weeks’ gestation.

Abbreviations: Caesarean, caesarean section; N/A, data not available.

### Medications

Most women were taking dual (52%) or single (37%) immunosuppression before conception, of which prednisolone (59%) and/or azathioprine (67%) were the most commonly used agents (Table 1). One unplanned pregnancy occurred while taking mycophenolate, which was subsequently stopped at 10 weeks’ gestation; no maternal or neonatal complications were noted. One woman had received a rituximab infusion 6 months before conception; this pregnancy ended with a neonatal complication of jaundice requiring ultraviolet therapy. Issues regarding immunosuppression nonadherence were identified in 10 (37%) pregnancies.

### Antenatal Surveillance of Cirrhosis

Of the 12 pregnancies occurring in women with liver cirrhosis, 9 (75%) underwent oesophageal‐gastro‐duodenoscopy (OGD), 5 of which found no evidence of varices, whilst 1 showed evidence of portal hypertensive gastropathy with no bleeding, and 3 demonstrated varices that required banding. Liver ultrasound was performed in 10 pregnancies (83%), none of which found any evidence of splenic artery aneurysms. Four pregnancies (33%) for which caesarean sections were being considered underwent pelvic magnetic resonance imaging, none of which found evidence of pelvic varices.

### Pregnancy Outcomes

The median gestation at delivery was 38 weeks (IQR 36‐39), with 30% ending in premature births (Table 3). Data regarding delivery method were available for n = 24 pregnancies, of which most of the deliveries were via caesarean section (63%). Pregnancies with cirrhosis were delivered via caesarean section in 73% (8 of 11) of cases, pregnancies with fibrosis in 33% (3 of 9), and pregnancies without cirrhosis or fibrosis in 100% (4 of 4). From our records, it was not possible to discern whether the caesarean sections had been performed due to patient choice or clinical need.

**TABLE 3:** Pregnancy Outcomes

	Total (n)	Statistic
Delivery method	24	
*Caesarean section*		15 (63%)
*Vaginal*		9 (38%)
Gestation at delivery (weeks)	27	38 (36‐39)
Premature birth	27	8 (30%)
Any pregnancy complication*	27	14 (52%)
Maternal complications^†^	27	
*Cholestasis of pregnancy*		4 (15%)
*Gestational diabetes, new onset*		2 (7%)
*Gestation diabetes, worsening*		1 (4%)
*Hypertension of pregnancy*		1 (4%)
*Placenta previa/retained placenta*		1 (4%)
*Antepartum hemorrhage/ruptured membranes*		1 (4%)
*Peripartum renal failure*		1 (4%)
Neonatal complications^†^	27	
*Fetal distress*		2 (7%)
*Jaundice*		1 (4%)
*Reduced fetal heartbeat*		1 (4%)
*Growth retardation*		1 (4%)
*Extended neonatal unit stay*		1 (4%)
Antenatal flare	25	3 (12%)
Postpartum flare	26	15 (58%)

Note: Data are reported as n (%) or median (IQR), as applicable.

* Pregnancies in which any of the listed maternal or neonatal complications occurred.

^†^ Not mutually exclusive.

The most commonly recorded maternal complication was cholestasis of pregnancy (15%), while the most common neonatal complication was fetal distress (7%). It was not possible to ascertain whether an antenatal flare occurred during two pregnancies, due to a lack of available blood tests. Of the remainder, an antenatal flare occurred during 12% of pregnancies (3 of 25), all of which also experienced a postpartum flare, and two had concerns about medication adherence noted. We were not able to carry out a full statistical analysis of antenatal flares due to the low incidence. Postpartum blood tests were unavailable for one pregnancy; hence, it could not be ascertained whether a postpartum flare had occurred. Of the remainder, 58% (15 of 26) developed a postpartum flare.

Associations between patient factors and the primary pregnancy outcomes of premature birth and postpartum flare were then assessed (Table 4). These analyses had low statistical power on account of the small sample size; hence, only large effects were detectable. Despite this, pregnancies in women with AIH type 2 were found to have a significantly higher rate of premature births than those with type 1 disease (67% vs. 19%, *P* = 0.044), with a near‐significant tendency for a higher rate of postpartum flare also observed (100% vs. 48%, *P* = 0.053). Additionally, although not significant, there was almost double the risk of prematurity (40% vs. 24%, *P* = 0.415) and postpartum flare (80% vs. 44%, *P* = 0.109) in pregnancies with issues surrounding medication nonadherence. The single patient with a twin pregnancy had developed a severe postpartum flare, with an ALT of 758 U/L and bilirubin of 102 µmol/L.

**TABLE 4:** Associations With Pregnancy Outcomes

	Premature Birth	Postpartum Flare
Age at conception (years)	*P* = 0.076*	*P* = 0.375*
*<30*	43% (6 of 14)	69% (9 of 13)
*30+*	15% (2 of 13)	46% (6 of 13)
Ethnicity	*P* = 1.000	*P* = 0.109
*White*	29% (5 of 17)	44% (7 of 16)
*Non‐White*	30% (3 of 10)	80% (8 of 10)
Previous pregnancies	*P* = 1.000	*P* = 0.428
*No*	29% (4 of 14)	46% (6 of 13)
*Yes*	31% (4 of 13)	69% (9 of 13)
AIH diagnosis to conception (years)	*P* = 0.665*	*P* = 0.229*
*<6*	25% (3/12)	58% (7/12)
*6+*	33% (5/15)	57% (8/14)
AIH type	P = 0.044	*P* = 0.053
*Type 1*	19% (4 of 21)	48% (10 of 21)
*Type 2*	67% (4 of 6)	100% (5 of 5)
Associated autoimmune disease^†^	*P* = 0.633	*P* = 0.658
*No*	35% (7 of 20)	53% (10 of 19)
*Yes*	14% (1 of 7)	71% (5 of 7)
Fibrosis/cirrhosis	*P* = 0.643	*P* = 0.342
*No*	40% (2 of 5)	60% (3 of 5)
*Fibrosis*	20% (2 of 10)	40% (4 of 10)
*Cirrhosis*	33% (4 of 12)	73% (8 of 11)
ANA	*P* = 0.087	*P* = 0.701
*Negative*	50% (6 of 12)	64% (7 of 11)
*Positive*	13% (2 of 15)	53% (8 of 15)
Anti‐smooth muscle antibody	*P* = 0.420	*P* = 1.000
*Negative*	38% (5 of 13)	58% (7 of 12)
*Positive*	21% (3 of 14)	57% (8 of 14)
SLA/LP antibodies	*P* = 0.374	*P* = 1.000
*Negative*	24% (4 of 17)	65% (11 of 17)
*Positive*	43% (3 of 7)	57% (4 of 7)
Ro‐52 antibodies	*P* = 1.000	*P* = 1.000
*Negative*	33% (2 of 6)	50% (3 of 6)
*Positive*	33% (2 of 6)	50% (3 of 6)
LKM blot	*P* = 1.000	*P* = 0.351
*Negative*	28% (5 of 18)	56% (10 of 18)
*Positive*	33% (2 of 6)	83% (5 of 6)
LKM antibodies	*P* = 0.281	*P* = 0.125
*Negative*	24% (5 of 21)	52% (11 of 21)
*Positive*	60% (3 of 5)	100% (4 of 4)
Medication nonadherence	*P* = 0.415	*P* = 0.109
*No*	24% (4 of 17)	44% (7 of 16)
*Yes*	40% (4 of 10)	80% (8 of 10)
Prepregnancy immunosuppression regime	*P* = 0.696	*P* = 0.692
*None/single*	25% (3 of 12)	50% (6 of 12)
*Dual/triple*	33% (5 of 15)	64% (9 of 14)

Note: Data are reported as % (n), and *P* values are from Fisher’s exact tests, unless stated otherwise. Bold *P* values indicate significance at *P* < 0.05.

* *P* values from Mann‐Whitney U tests, comparing the exact numbers of years between those with versus without outcomes.

^†^ Other than type 1 diabetes mellitus.

Abbreviations: LC‐1, liver cytosol antigen type 1; f‐actin, filamentous actin.

### Trends in ALT and IgG

**FIG. 1:**
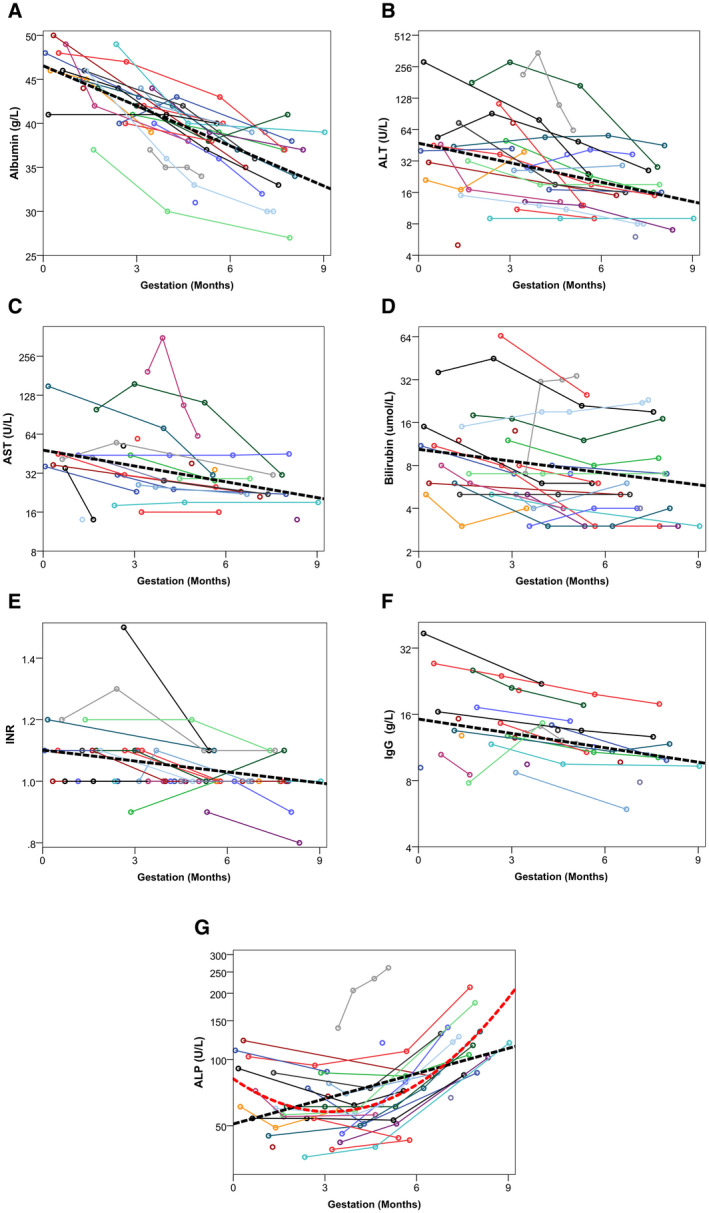
Trends in blood markers over the antenatal period. Solid lines represent individual pregnancy trajectories. Broken black lines are trends for the cohort as a whole and are derived from the models reported in Table 5. The *y* axes use logarithmic scales for all markers, except for albumin and INR, to improve scaling. The plot of ALP includes an additional broken red line, which is based on a polynomial model that includes a gestation‐squared term. (A) trends in albumin (g/L), (B) trends in ALT (U/L), (C) trends in AST (U/L), (D) trends in bilirubin (umol/L), (E) trends in INR, (F) trends in IgG (g/L), (G) trends in ALP (U/L).

**FIG. 2:**
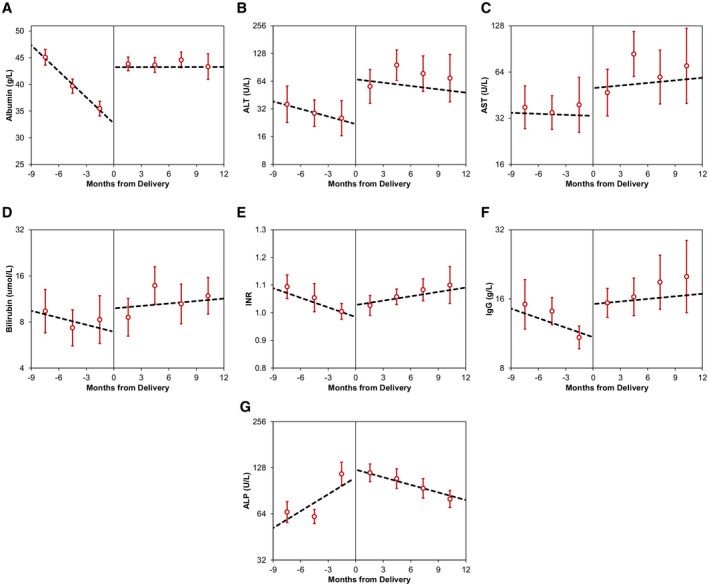
Trends in blood markers in the antenatal versus postpartum periods. The horizontal line represents the point of delivery; hence, the antenatal period is to the left, and the postpartum period is to the right. Points represent the arithmetic or geometric mean of all samples taken within each three monthly interval, and are plotted at the midpoint of the interval. Error bars indicate 95% CIs. Broken lines are derived from the models reported in Table 6. (A) trends in albumin (g/L), (B) trends in ALT (U/L), (C) trends in AST (U/L), (D) trends in bilirubin (umol/L), (E) trends in INR, (F) trends in IgG (g/L), (G) trends in ALP (U/L).

A total of n = 71 antenatal blood tests were performed in n = 25 pregnancies, with a median of three tests per pregnancy (range: 1‐5). In addition to the antenatal samples, n = 107 samples were collected from n = 26 of the pregnancies over the postpartum period, with a median of four per pregnancy (range: 1‐11). In each case, data were not available for all markers from each blood test. Initially, models were produced using only data from the antenatal period (Table 5 and Fig. 1); these were then extended to include postpartum samples (Table 6 and Fig. 2), to allow comparisons between the two periods.

**TABLE 5:** Trends in Blood Markers Over the Antenatal Period

	Samples (n)	Gradient per Month (95% CI)	*P* Value
Albumin (g/L)	70	−1.52 (−1.72, −1.31)	**<0.001**
ALP (U/L)*	70	9.2% (6.4%, 12.0%)	**<0.001**
ALT (U/L)*	69	−13.3% (−17.1%, 9.4%)	**<0.001**
AST (U/L)*	53	−8.9% (−12.4%, −5.3%)	**<0.001**
Bilirubin (umol/L)*	69	−6.2% (−9.0%, −3.2%)	**<0.001**
INR	60	−0.012 (−0.018, −0.006)	**<0.001**
IgG (g/L)*	48	−4.9% (−6.4%, −3.4%)	**<0.001**

Note: Results are from general linear models with the pregnancy ID and gestation of the sample as covariates. Bold *P* values indicate significance at *P* < 0.05.

* Blood markers followed skewed distributions, and therefore log_10_‐transformed before the analysis. The resulting coefficients were then antilogged and converted into percentage changes per month.

**TABLE 6:** Trends in Blood Markers Over the Antenatal Versus Postpartum Periods

	Antenatal			Postpartum			Change in Gradient *P* Value^†^	Step‐Change	
	(n)*	Gradient per Month (95% CI)	*P* Value	(n)*	Gradient per Month (95% CI)	*P* Value		Coefficient (95% CI)	*P* Value
Albumin (g/L)	70	−1.63 (−1.88, −1.38)	**<0.001**	104	0.00 (−0.16, 0.17)	0.974	**<0.001**	10.55 (9.02, 12.08)	**<0.001**
ALP (U/L)	70	8.8% (6.2%, 11.6%)	**<0.001**	104	−3.7% (−5.3%, −2.1%)	**<0.001**	**<0.001**	12.4% (−3.6%, 31.0%)	0.136
ALT (U/L)	69	−6.1% (−12.0%, 0.3%)	0.060	102	−2.7% (−6.9%, 1.7%)	0.219	0.383	205.7% (103.3%, 359.6%)	**<0.001**
AST (U/L)	53	−0.5% (−6.8%, 6.2%)	0.878	85	1.3% (−2.8%, 5.6%)	0.538	0.647	51.7% (1.0%, 127.7%)	**0.045**
Bilirubin (umol/L)	69	−3.4% (−7.7%, 1.0%)	0.127	104	1.2% (−1.8%, 4.3%)	0.425	0.089	42.1% (7.7%, 87.3%)	**0.013**
INR	60	−0.012 (−0.017, −0.006)	**<0.001**	94	0.005 (0.002, 0.009)	**0.006**	**<0.001**	0.043 (0.010, 0.077)	**0.012**
IgG (g/L)	48	−3.1% (−4.8%, −1.4%)	**<0.001**	66	0.9% (−0.3%, 2.0%)	0.141	**<0.001**	39.4% (25.2%, 55.2%)	**<0.001**

Note: Results are from interrupted time‐series general linear models, which included the following covariates: pregnancy ID, timing of the sample (relative to delivery), antenatal versus postpartum sample, and an interaction term between the latter two factors. The model was then evaluated to estimate the gradients in the antenatal and postpartum periods, as well as the step change that occurred directly after delivery. Blood markers with skewed distributions were log_10_‐transformed before the analysis; the resulting coefficients were then antilogged and converted into percentage differences, to simplify interpretation. Bold *P* values indicate significance at *P* < 0.05.

* The total number of antenatal/postpartum samples included in the analysis.

^†^ *P* value of the interaction term, representing a comparison of the antenatal and postpartum gradients.

During the antenatal period, markers of disease activity were found to decline significantly, with ALT decreasing by an average of 13.3% per month (95% confidence interval [CI]: 9.4%‐17.1%, *P* < 0.001) and IgG by 4.9% per month (95% CI: 3.4%‐6.4%, *P* < 0.001). After delivery, both markers saw a significant step‐change increase, with ALT levels tripling (205.7% increase, 95% CI: 103.3%‐359.6%, *P* < 0.001) and IgG increasing by 39.4% (95% CI: 25.2%‐55.2%, *P* < 0.001). Both markers then remained relatively stable for the 12 months postpartum, with no significant gradient detected in either ALT (*P* = 0.219) or IgG (*P* = 0.141).

### Trends in Other Biomarkers

Similar significant antenatal declines were also observed in albumin, AST, bilirubin, and INR (all *P* < 0.001; Table 5 and Fig. 1). The initial model of ALP found levels to increase significantly over the antenatal period (*P* < 0.001). However, this model was found to have suboptimal fit, and so the analysis was repeated with a quadratic model, which is visualized in Fig. 1G. This found ALP levels to decline early in the pregnancy, reaching a trough at approximately 4 months gestation, after which levels began to increase.

After delivery, significant step‐change increases were observed in albumin (*P* < 0.001), AST (*P* = 0.045), bilirubin (*P* = 0.013), and INR (*P* = 0.012). No significant step change was observed for ALP (*P* = 0.136), with this marker instead showing an inverted “V‐shaped” trend, increasing by 8.8% per month (95% CI: 6.2%‐11.6%, *P* < 0.001) in the antenatal period, before this reversed to a 3.7% per month decline (95% CI: 2.1%‐5.3%, *P* < 0.001) in the postpartum period. INR also showed a “V‐shaped” trend, with the gradient changing from a 0.012 per month decline (95% CI: 0.006‐0.017, *P* < 0.001) to a 0.005 per month increase (95% CI: 0.002‐0.009, *P* = 0.006) between the two periods.

### Blood Markers in Repeat Pregnancies

**FIG. 3:**
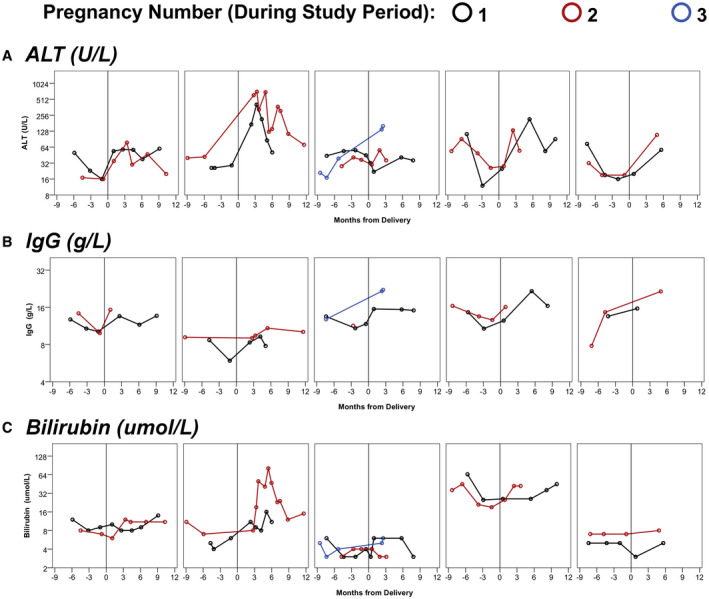
Trends in blood markers for patients with repeat pregnancies. Each plot represents a single patient, with data for all pregnancies included in the cohort. One patient with multiple pregnancies was excluded, as she had no antenatal samples. (A) trends in ALT (U/L), (B) trends in IgG (g/L), (C) bilirubin (umol/L).

The n = 6 women with data from multiple pregnancies were then further assessed. Of these, two had additional pregnancies before the study period, for which data were not available. Of the 3 women who had postpartum flares in their first pregnancy during the study period, 2 (66%) had a flare in their second pregnancy. For the 3 without a postpartum flare in their first pregnancy, 2 (66%) had a flare in their second pregnancy. Figure 3 shows the trajectories of IgG, ALT, and bilirubin for the n = 5 patients who had samples from both the antenatal and postpartum periods.

## Discussion

With the improvement of multidisciplinary care among hepatologists, obstetricians, and clinical nurse specialists in AIH, pregnancy is becoming increasingly common in patients with this rare disease.^(^ ^5^, ^7^ ^)^

Using an interrupted time‐series approach, we have shown that during pregnancies in women with AIH, ALT and IgG decline significantly with gestation, followed by a significant step‐change increase immediately after delivery. Remission of AIH during pregnancy is well documented, having been reported in several previous studies.^(^ ^4^, ^8^, ^9^, ^10^ ^)^ Despite the course of the disease appearing to enter a period of remission during pregnancy, cases of flare during the gestation period have been reported.^(^ ^13^ ^)^ The incidence of postpartum flares in AIH has been described in 13% to 52% of pregnancies.^(^ ^7^, ^8^, ^10^, ^11^, ^13^ ^)^ In accordance with previous case series, in our cohort, pregnancy was associated with reductions in disease activity in most cases, with antenatal flares in disease activity occurring in only 12% of cases. This was followed by a high incidence of postpartum flares, which occurred in over half of the pregnancies. For the subgroup of women with multiple pregnancies, blood markers appeared to follow similar trends across all pregnancies. However, due to the limited quantity of data in this subgroup, further investigation, including immunological studies, would be warranted to quantify the associations between disease activity in repeated pregnancies.

We did not confirm the association between the presence of SLA/LP and Ro/SSA antibodies and adverse pregnancy outcomes in AIH, as found in a previous study.^(^ ^11^ ^)^ However, our data show for the first time a correlation between type 2 AIH and postpartum flares. Recently, the subclassification of AIH into type 1 and type 2 has been debated, because it fails to discriminate phenotypic and prognostic differences.^(^ ^14^ ^)^ Despite this, it is well accepted that type 2 AIH has a more aggressive onset in children, with a high rate of fulminant hepatitis.^(^ ^15^ ^)^ Data on long‐term outcomes in these patients are currently lacking, due to the difficulty in maintaining follow‐up after transition to adult care. Our data suggest that there is an augmented risk of disease relapse after pregnancy in patients with type 2 AIH, although this did not reach statistical significance, meaning that further multicenter studies would be required to confirm this finding. A significantly increased rate of preterm birth was also observed in patients with type 2 AIH. The evaluation of miscarriage and stillbirth was beyond the scope of our study. Nonetheless, it would be interesting to evaluate the rate of pregnancy related outcomes in patients with type 2 AIH.

Nonadherence with medications was a concern in 37% of pregnancies in our cohort. Although issues surrounding medication adherence in AIH have been reported in children,^(^ ^16^ ^)^ patients with AIH generally report their medication adherence as “good.”^(^ ^17^ ^)^ Concerns regarding adherence have previously been reported among patients with other long‐term conditions during pregnancy, reporting rates similar to our findings, with 36.2% of patients having low adherence to medication regimes.^(^ ^18^ ^)^ We found that there was a trend toward more premature births (40% vs. 24%) and postpartum flares (80% vs. 44%) in pregnancies in which medication adherence was an issue, although neither of these differences reached statistical significance. Additionally, 66% of antenatal flares occurred in pregnancies with medication‐adherence concerns. An exploration of the attitudes of women with AIH regarding the use of immunosuppression during pregnancy would be useful, to fully understand why there are issues with adherence, and how to devise strategies to overcome them.

Twelve of the pregnancies in our study occurred in the presence of liver cirrhosis, which has previously been shown to increase obstetric risks.^(^ ^6^ ^)^ One serious complication is variceal bleeding, occurring from the second trimester onward, and during labor.^(^ ^6^, ^19^ ^)^ Variceal bleeding carries a high rate of mortality and morbidity in the context of liver cirrhosis.^(^ ^6^ ^)^ Most of our patients with cirrhosis underwent OGD for variceal surveillance, and, importantly, none of our patients experienced any instances of variceal bleeding during pregnancy or labor. In our study, patients with cirrhosis underwent caesarean section in 73% of pregnancies. This is higher than the average rate for Europe^(^ ^20^ ^)^; however, it has previously been reported that patients with liver cirrhosis have higher rates of caesarean sections.^(^ ^21^, ^22^ ^)^ In patients with cirrhosis, the choice of delivery method should be based on obstetric indications, as there is limited evidence to recommend a preferred method with these patients.^(^ ^23^ ^)^

Our study suffers from being a retrospective and relatively small study, with potential selection bias and missing data confounding the analysis. The small sample size resulted in low statistical power, particularly when analyzing associations with pregnancy outcomes (postpartum flare and premature birth). Additionally, we did not include miscarriages and stillbirths, as the numbers of these were small and, hence, it was not possible to compare these to pregnancies ending in live births. However, there may be a difference in blood marker trends among live births, stillbirths and miscarriages, and this is a topic that we would like to explore in the future.

In conclusion, biochemical and immunological remission of AIH occurs during pregnancy, with albumin, ALT, AST, bilirubin, and IgG declining significantly as gestation progresses. Postpartum flare is common in AIH, with ALT and IgG showing significant step‐change increases after delivery.

The risk of both premature birth and postpartum flare appears to be higher in type 2 AIH and may be increased in patients with nonadherence to medication. Multidisciplinary care between the obstetric and liver teams with regular meetings to discuss maternal and fetal progress is crucial for the management of pregnant patients with AIH. As twin pregnancy was associated with a severe flare‐up of AIH, these patients should be monitored closely in the postpartum period. Exploration of the immunological mechanism of remission of AIH during pregnancy and immune tolerance breakdown during the postpartum period with both blood and placental tissue is warranted, to predict and prevent postpartum flare. Further evaluation of the attitudes of patients with AIH toward the importance of medication adherence of immunosuppression during pregnancy can be addressed during prepregnancy counseling.
